# Comparison of the efficacy of vocal training and vocal microsurgery in patients with early vocal fold polyp^☆^

**DOI:** 10.1016/j.bjorl.2018.03.014

**Published:** 2018-05-09

**Authors:** Hanqing Wang, Pan Zhuge, Huihua You, Yulan Zhang, Zhifeng Zhang

**Affiliations:** Jinhua Central Hospital, Department of Otolaryngology, Jinhua, China

**Keywords:** Early vocal fold polyp, Voice training, Voice microsurgery, Laryngostroboscopy, Voice handicap index, Dysphonia severity index, Pólipo de prega vocal incipiente, Treinamento de voz, Microcirurgia da voz, Laringo-estroboscopia, Índice de desvantagem vocal, Índice de severidade de disfonia

## Abstract

**Introduction:**

Vocal fold polyp is a benign proliferative disease in the superficial lamina propria of the vocal fold, and vocal microsurgery can improve the voice quality of patients with vocal fold polyp. In preliminary studies, we found that vocal training could improve the vocal quality of patients with early vocal fold polyp.

**Objective:**

This study aimed to compare the efficacies of vocal training and vocal microsurgery in patients with early vocal fold polyp.

**Methods:**

A total of 38 patients with early vocal fold polyp underwent 3 months of vocal training (VT group); another 31 patients with early vocal fold polyp underwent vocal microsurgery (VM group). All subjects were assessed using laryngostroboscopy, voice handicap index, and dysphonia severity index, and the efficacies of vocal training and vocal microsurgery were compared.

**Results:**

The cure rates of vocal training and vocal microsurgery were 31.6% (12/38) and 100% (31/31), respectively. The intragroup paired-sample *t*-test showed that the post treatment vocal handicap index, maximum phonation time, highest frequency (F0-high), lowest intensity (I-low), and dysphonia severity index in both the VT and VM groups were better than those before treatment, except for the jitter value. The intergroup independent-sample *t*-test revealed that the emotional values of vocal handicap index (*t* =  − 2.22, *p* = 0.03), maximum phonation time (*t* = 2.54, *p* = 0.013), jitter (*t* =  − 2.11, *p* = 0.03), and dysphonia severity index (*t* = 3.24, *p* = 0.002) in the VT group were better than those in the VM group.

**Conclusions:**

Both, vocal training and vocal microsurgery could improve the voice quality of patients with early vocal fold polyp, and these methods present different advantages.

## Resumo

**Introdução:**

O pólipo de prega vocal é uma doença proliferativa benigna da camada superficial da lâmina própria da prega vocal, e a microfonocirurgia pode melhorar a qualidade vocal desses pacientes. Em estudos preliminares, observamos que o treinamento vocal era capaz de melhorar a qualidade vocal de pacientes com pólipo incipiente de prega vocal.

**Objetivo:**

Este estudo teve como objetivo comparar a eficiência entre treinamento vocal e microfonocirurgia em pacientes com pólipo incipiente de prega vocal.

**Método:**

Um total de 38 pacientes com pólipo incipiente de prega vocal foram submetidos a três meses de treinamento vocal (grupo TV); outros 31 pacientes foram submetidos à microfonocirurgia (grupo MC). Todos os indivíduos foram avaliados por meio de laringoestroboscopia, índice de desvantagem vocal e índice de severidade da disfonia, e a eficácia entre treinamento vocal e microfonocirurgia foi comparada.

**Resultados:**

As taxas de cura do treinamento vocal e da microfonocirurgia foram de 31,6% (12/38) e 100% (31/31), respectivamente. O teste *t* para amostras pareadas intragrupo mostrou que o índice de desvantagem vocal pós-tratamento, tempo máximo de fonação, frequência máxima, intensidade mínima e índice de severidade da disfonia nos grupos TV e MC foram melhores do que aqueles antes do tratamento, exceto pelo valor do *jitter*. O teste *t* para amostras independentes intergrupos revelou que o valor emocional do índice de desvantagem vocal (t = -2,22, *p* = 0,03), tempo máximo de fonação (t = 2,54, *p* = 0,013), *jitter* (t = -2,11, *p* = 0,03) e índice de severidade da disfonia (t = 3,24, *p* = 0,002) no grupo TV foram melhores do que os do grupo MC.

**Conclusões:**

Tanto o treinamento vocal quanto a microfonocirurgia podem melhorar a qualidade da voz de pacientes com pólipo incipiente de prega vocal e esses métodos apresentam diferentes vantagens.

## Introduction

Vocal fold polyp (VFP) is a benign proliferative disease in the superficial lamina propria of the vocal fold; however, there is no consensus regarding the selection of treatment options for this condition. Related studies have indicated that vocal microsurgery (VM) could improve the voice quality of patients with VFP, with a low postoperative recurrence rate.^1^, ^2^, ^3^ However, surgery entails economic burdens, surgical risks, and general anesthesia risks,^4^ which might be the main reasons why some patients refuse surgery. Vocal therapy includes vocal training (VT) and vocal health education, and studies have shown that vocal therapy has certain therapeutic effects on VFP.^5^, ^6^ Compared with vocal surgery, vocal therapy takes longer. Furthermore, in this treatment approach, the patients’ compliance may be influenced by several factors such as low educational level and lack of medical knowledge of the patient; insufficient experience, inadequate training time, and ineffective follow-up of the physician; long patient waiting time for appointment; inconvenient travel conditions; and conflicts between the training time and the work of the patient. Failure of compliance might result in adverse impacts on the therapeutic effects and even cause patients to terminate their vocal therapy sessions.^7^, ^8^, ^9^

The pathophysiology of VFP includes primary bleeding, edema, and fibrin deposition.^10^ Dursun et al.^11^ found that compared with patients with large polyps, the jitter value of patients with small polyps was significantly lower. Through multivariate analysis, Cho et al.^12^ considered that among such clinical factors as VFP size, location, site of origin, color, and laryngopharyngeal reflux, the polyp size was the only factor related to vocal quality. In preliminary studies, we applied the Voice Handicap Index (VHI) and Dysphonia Severity Index (DSI) to assess the vocal quality of 88 patients with early VFP (EVFP), and we found that EVFP manifested as various degrees of subjective and objective vocal disorders. We also found that VT could improve the vocal quality of these patients, thus confirming its value in treating EVFP.^13^

The main purpose of this study was to compare the clinical efficacies of VT and VM in patients with EVFP, and to summarize our clinical experience.

## Materials and methods

### Clinical data

The study included patients with EVFP treated at the Department of Otolaryngology, Jinhua Central Hospital, from September 2013 to February 2015. The inclusion criteria were as follows: (1) Main complaint of hoarseness and disease duration of <6 months; (2) Laryngoscopy results showing that the polyp was located at the one-third junction of the anteromedian vocal fold, appearing as fusiform translucent small bump(s) (diameter: less than one-fourth of the vocal fold).^12^ The exclusion criteria included a polyp diameter greater than one-fourth of the vocal fold, and pedunculated VFP, vocal fold tumor, vocal nodule(s), or Reinke's edema. Thirty-eight patients chose to undergo VT and vocal health education, and were included in the VT group (group A). Thirty-one patients selected VM followed by postoperative vocal health education and were included in the VM group (group B). All study subjects signed an informed consent form. This study was conducted with approval from the Ethics Committee of Jinhua Central Hospital (0579-82552825). Written informed consent was obtained from all participants.

There was no statistically significant difference in sex, age, or occupational vocal usage (Table 1) among the two groups. Subjects with occupational vocal usage included teachers, salespersons, counselors, and tour guides, and their working durations were all > 6 months.

**Table 1:** Clinical factors among VT, VM and control groups.

Parameter	VT group (*n* = 38)	VM group (*n* = 31)	*p*
*Gender, n (%)*			0.254
M	12 (31.6%)	6 (19.4%)	
F	26 (68.4%)	25 (80.6%)	

*Age (mean* *±* *SD; y)*	39.71 ± 8.71	36.58 ± 9.65	0.162
*Disease duration (mean* *±* *SD; m)*	4.46 ± 1.07	4.74 ± 1.09	0.285
*Occupational vocal usage, n (%)*			0.962
Yes	10 (26.3%)	8 (25.8%)	
No	28 (73.7%)	23(74.2%)	

### Laryngostroboscopy

The XION laryngostroboscopy system (XION, Germany) was used for the inspection. The subjects were seated in a quiet environment and treated with 1% tetracaine spray three times to anesthetize the throat mucosa; subsequently, the subjects were asked to relax and breathe calmly. The lens was then inserted into the throat, adjacent to the posterior pharyngeal wall and parallel to the vocal fold level. The subjects were asked to pronounce the letter “I,” and the polyp size, location, vibration symmetry, period, amplitude, closure, and mucous membrane fluctuations were observed, recorded, and evaluated by the inspector.

### Self-subjective assessment

A medical staff was assigned to explain the meaning of the study to the subjects, and the subjects scored the Physiological (P), Functional (F), and Emotional (E) sections of the questionnaire by using the Chinese version of VHI, without assistance. Each section included 10 questions, and the options represented the frequency of occurrence of the corresponding item, as follows: 0 point, “never”; 1 point, “rarely”; 2 points, “sometimes”; 3 points, “regularly”; and 4 points, “always”. The score of each section was the sum of the scores for the 10 questions, ranging from 0 to 40 points; Total score (T) was the sum of the scores of the three sections, ranging from 0 to 120 points. The higher the score in one section, the greater the impact of this section on the study subject. The higher the T, the more severe the VHI according to the subject's own subjective assessment.^14^

### Objective acoustics, aerodynamics evaluation, and DSI calculation

The evaluation was performed in one voice test room by using the DiVAS voice analysis software (XION, Germany). Each study subject wore a headset microphone, with the microphone probe 30 cm away from the subject's mouth. After relaxing and breathing calmly, each subject was tested for the Maximum Phonation Time (MPT), jitter, Highest Frequency (F0-high), and Lowest Intensity (I-low), and the DSI score was calculated.

*MPT test*: After breathing deeply, the subjects were told to continuously pronounce the vowel “a” with a self-felt comfortable tone and intensity for as long as possible. The test was repeated three times and the longest vocal sample was used.

*Jitter test*: The subjects were told to pronounce the vowel “a” with a self-felt comfortable tone and intensity for 3 s. This test was repeated three times. The jitter value of each pronunciation within 0.5–1.5 s was assessed at each test, and the average value was used.

*F0-high and I-low test*: The subjects were told to pronounce the vowel “a” with a self-felt comfortable tone and intensity, and this specific comfortable tone and intensity were recorded as the base points. The subjects were then told to pronounce the vowel “a” with as high a tone and intensity as possible, and then as low as possible, gradually. The averages of F0-high and I-low of the three tests were used.

The DSI score was calculated as follows:

DSI=0.13×MPT+0.0053×F0-high−0.26×I-low−1.18×jitter+12.4.

### Surgical methods

Patients in the VM group underwent surgery performed by the same experienced otorhinolaryngologist under general anesthesia and a microscope. The polyp was removed, and the microstructures of the vocal fold were retained as much as possible.^15^, ^16^, ^17^, ^18^

### Vocal therapy protocol

The patients in the VT group received 3 months of VT and vocal health education from one experienced otorhinolaryngologist. Each training course was approximately 60–90 min long, and conducted once every 2 weeks. The VT contents included the following activities: (1) relaxation training, 2) breathing training, (3) vocal posture, (4) balance of vocal organs, and (5) vocal acoustic training. The contents of vocal health education included the prevention of vocal misuse and abuse, and universal throat health knowledge.^19^, ^20^, ^21^, ^22^ All patients received the training materials prepared by us to facilitate their continuous practice at home. Additionally, we also followed-up the patients via telephone to determine their exercise progress and answer their questions. Patients in the VM group were given postoperative vocal health education.

### Efficacy evaluation

The VT group was reevaluated 1 month after the VT (the vocal training time was three months), and the VM group was reevaluated 4 months after the surgery. Both groups were reevaluated with laryngostroboscopy, with vocal polyp disappearance as the cure criterion, along with reevaluation of VHI and retesting of MPT, jitter, F0-high, and I-low to recalculate the DSI.

### Statistical analysis

If the data showed normality and homogeneity of variance, they were expressed as mean ± standard deviation, and intergroup data were analyzed using the *t*-test. If the data did not show normality and homogeneity of variance, they were expressed as median and quartile, and analyzed using the rank sum test (*p* < 0.05 was considered statistically significant).

## Results

### Vocal assessment

Laryngostroboscopy revealed that the VFPs in the VT and VM groups were located at the one-third junction of the anteromedian vocal fold. There was no statistically significant difference in VHI and DSI in both the VT and VM groups before the treatment (Table 2).

**Table 2:** Vocal assessment of the three groups before the treatment.

Parameter	VT group ($\bar{x}\pm s$)	VM group ($\bar{x}\pm s$)	*p*
F	9.08 ± 6.94	9.26 ± 8.1	0.922
P	16.03 ± 9.47	18.55 ± 8.57	0.255
E	7.13 ± 8.14	9.13 ± 10.03	0.364
T	30.92 ± 22.48	36.61 ± 25.27	0.326
MPT (s)	16.62 ± 3.47	17.28 ± 3.52	0.441
Jitter (%)	1.41 ± 0.73	1.51 ± 0.63	0.561
F_0_-High (Hz)	397.66 ± 47.26	375.9 ± 59.35	0.095
I-Low (dB)	56.71 ± 3.42	58.06 ± 4.03	0.136
DSI	0.28 ± 1.18	−0.22 ± 1.43	0.117

### Treatment efficacies

The follow-up examination revealed that the cure rates of the VT and VM groups were 31.6% (12/38) and 100% (31/31), respectively. The intragroup paired-sample *t*-test showed that the post treatment VHI, MPT, F0-high, I-low, and DSI in both treatment groups showed significant improvement as compared with those before treatment, except for the jitter value (Table 3). Among the 26 uncured patients in the VT group, 18 patients selected surgical treatment, whereas the remaining 8 patients selected vocal therapy sequentially.

**Table 3:** Vocal assessment of VT and VM groups before and after the treatment.

Parameter	Before treatment ($\bar{x}\pm s$)	Follow-up ($\bar{x}\pm s$)	*t*	*p*
*VT group*				
F	9.08 ± 6.94	5.66 ± 5.4	7.68	0.000
P	16.03 ± 9.47	11.29 ± 8.09	7.47	0.000
E	7.13 ± 8.14	3.95 ± 4.89	5.45	0.000
T	30.92 ± 22.48	20.74 ± 15.93	6.01	0.000
MPT (s)	16.62 ± 3.47	20.09 ± 3.58	−9.11	0.000
Jitter (%)	1.41 ± 0.73	1.13 ± 0.41	4.03	0.000
F_0_-High (Hz)	397.66 ± 47.26	410.33 ± 49.76	−5.71	0.000
I-Low (dB)	56.71 ± 3.42	54.89 ± 2.9	5.05	0.000
DSI	0.28 ± 1.18	1.63 ± 0.79	−9.38	0.000

*VM group*				
F	9.26 ± 8.1	6.26 ± 6.35	6.11	0.000
P	18.55 ± 8.57	12.84 ± 6.81	8.51	0.000
E	9.13 ± 10.03	7.77 ± 9.16	6.45	0.000
T	36.61 ± 25.27	26.87 ± 21.63	8.27	0.000
MPT (s)	17.28 ± 3.52	17.78 ± 3.99	−2.66	0.012
Jitter (%)	1.51 ± 0.63	1.38 ± 0.55	3.96	0.000
F_0_-High (Hz)	375.9 ± 59.35	412.59 ± 45.97	−5.48	0.000
I-Low (dB)	58.06 ± 4.03	55.35 ± 4	8.79	0.000
DSI	−0.22 ± 1.43	0.78 ± 1.34	−4.12	0.000

### Efficacy comparison

The Mann–Whitney *U*-test revealed that the cure rate of the VM group was better than that of the VT group (*Z* = −5.792, *p* = 0.000). The intergroup independent-sample *t*-test revealed that the E-value, MPT, jitter value, and DSI showed greater improvement in the VT group than in the VM group (Table 4).

**Table 4:** Efficacy comparison between VT and VM groups after the treatment.

Parameter	VT ($\bar{x}\pm s$)	VM ($\bar{x}\pm s$)	*t*	*p*
F	5.66 ± 5.4	6.26 ± 6.35	−0.42	0.673
P	11.29 ± 8.09	12.84 ± 6.81	−0.85	0.399
E	3.95 ± 4.89	7.77 ± 9.16	−2.22	0.03
T	20.74 ± 15.93	26.87 ± 21.63	−1.37	0.175
MPT (s)	20.09 ± 3.58	17.78 ± 3.99	2.54	0.013
Jitter (%)	1.13 ± 0.41	1.38 ± 0.55	−2.11	0.038
F_0_-High (Hz)	410.33 ± 49.76	412.59 ± 45.97	1.85	0.068
I-Low (dB)	56.71 ± 3.42	55.35 ± 4	−0.55	0.582
DSI	1.63 ± 0.79	0.78 ± 1.34	3.24	0.002

## Discussion

Previous studies on the treatment of VFP mainly focused on VM, and related reports proved its effectiveness for VFP.^15^, ^16^, ^17^, ^18^ For patients unwilling to undergo VM for various reasons, VT might be a suitable alternative treatment. Some researchers believe that smaller VFPs are associated with better results of VT and health education.^23^ Currently, there has been no report comparing VT and VM in EVFP. Our findings suggest that the post treatment vocal quality of the two treatment groups improved by various degrees, which suggests that both VT and VM were effective treatment methods for EVFP.

The differences in the principles and characteristics of the two described treatment methods should also be recognized. By removing diseased tissues, VM could effectively and quickly resolve the weakening of the mucosal wave caused by cladding stiffness, as well as other conditions such as polyp-induced glottis incompetence; thus, it improves the patients’ postoperative voice quality. However, VM would undoubtedly entail economic burdens and surgical risks to patients. Furthermore, surgery alone cannot correct the patients’ detrimental vocal habits. The application of vocal education would help patients gain vocal health knowledge, thus helping them avoid vocal misuse and abuse, reducing bad vocal behaviors caused by persistent vocal mucosa vibration trauma, and creating conditions for absorption and healing of VFPs. Through continuous practice, the balance among voice-related organs could be established. Therefore, voicing could be internalized into overall behaviors and activities, and proper voicing practices could be learned to improve patients’ objective pronunciation quality, consolidate the therapeutic effects, and prevent VFP recurrence. Therefore, the treatment helps patients clearly understand their own vocal problems, avoid anxiety related to vocal quality disorders, feel their own voice improvements, and establish a rational treatment expectation. Furthermore, this helps in improving the patients’ self-assessment of subjective vocal disorder. Owing to the differences in cultures, customs, habits, educational levels, and health-care levels in different regions and countries – especially the difference in phoniatrics – some Chinese doctors and patients have not understood the therapeutic values of VT and vocal health yet, and this treatment option is worthy of receiving large attention. Ju et al.^24^ systematically studied the changes in acoustics, aerodynamics, and patient self-evaluation of vocal disorder in patients with VFP who received VT after VM, and found that VT could improve the patients’ self-evaluation of their post-VM vocal disorder. Petrovic-Lazic et al.^25^ found that the analyzed acoustic parameters of patients with VFPs were improved after phonomicrosurgery and VT, and tended to approach the values of the control group. This provided good clinical evidence and guidance for a reasonable application of VM and VT.

Our results showed that the improvements of MPT, jitter, DSI, and E-values in the VT group were better than that in the VM group. MPT mainly reflects the patients’ ability to control their own vocal airflow, and jitter reflects the small rapid changes in fundamental vibration frequencies during the vocal phonating process, which could reflect the vocal vibration stability to some extent because it is influenced by vocal fold quality, tension, biomechanical characteristics, innervations, and other factors. We believe that VT could help improve the patients’ ability to control their vocal airflow and vocal vibration stability, thus showing better DSI evaluation levels. VHI is a questionnaire composed of functional, emotional, and physiological sections, in which the E-value (emotional section) describes vocal diseases-related emotional responses, and the assessment results might be influenced by each patient's character, disease duration, social status, educational level, or vocal usage in the social setting.^26^ After VT, patients with VFP could understand the characteristics of their diseases in a better manner. Moreover, after establishing the correct voicing patterns, patients could regain comfort during their verbal expression, experience the joy of verbal communication, and calm the mental anxiety caused by voice quality disorders. This may explain why, in our study, the patients applied vocal therapy achieved significantly improved E-values than those undergoing surgery. Martines et al.^27^ also indicated that voice therapy is effective to improve voice quality and to early detect and help reduce anxiety and depression symptoms. Such similar findings suggest the important clinical value of vocal therapy. On the other hand, our results showed that when the cure standard was the disappearance of VFP under the laryngoscope, the cure rate of the VM group was much better than that of the VT group. When physicians and patients select the treatment method, the cure rate is an important reference index. For the patients with vocal cord polyps disappear as the treatment target, surgery seems to be the preferred method. Our research summarizes and compares the subjective and objective vocal changes of these two treatment methods, as well as the cure rate, so it can provide a reference for patients to choose the treatment method that suits their own needs.

## Conclusion

Although both VM and VT could effectively improve the vocal quality of patients with EVFP, they represent different treatment benefits. When physicians and patients select the appropriate treatment method, they should fully understand the principles and characteristics of VT and VM. It may be desirable to select a treatment approach based on the patients’ own demands, as this would help establish reasonable treatment expectations. The limit of our research is that the sample number is not enough, and the follow-up time can be longer. The main concern refers to the possibility of selection bias, as the treatment groups where not randomized, nor stratified. As the patients were allowed for cross treatment arms after treatment, an RCT could be an interesting possibility to address this study question with less biased results. Further research needs to be carried out targeting these points.

## Conflicts of interest

The authors declare no conflicts of interest.
